# A randomized controlled trial of ovarian reserve preservation and hemostasis during ovarian cystectomy

**DOI:** 10.1038/s41598-021-87965-7

**Published:** 2021-04-19

**Authors:** Soo Jin Park, Aeran Seol, Nara Lee, Seungmee Lee, Hee Seung Kim, PRAHA Study Group, Aeran Seol, Eunji Lee, Ga Won Yim, Haerin Paik, Hee Seung Kim, Hyunji Lim, Jae-Weon Kim, Jaehee Mun, Junhwan Kim, Nara Lee, Seungmee Lee, Soo Jin Park

**Affiliations:** 1 Department of Obstetrics and Gynecology, Seoul National University College of Medicine, 101 Daehak-Ro Jongno-Gu, Seoul, 03080 Republic of Korea; 2 Department of Obstetrics and Gynecology, CHA Gangnam Medical Center, CHA University School of Medicine, Seoul, 06135 Republic of Korea; 3 Department of Obstetrics and Gynecology, Keimyung University School of Medicine, Daegu, 41931 Republic of Korea; 4 Department of Obstetrics and Gynecology, Dongguk University Ilsan Hospital, Goyang, Republic of Korea

**Keywords:** Endocrine reproductive disorders, Cysts

## Abstract

The preservation of ovarian reserve during laparoendoscopic single-site (LESS) ovarian cystectomy is crucial for reproductive-age women. This study was a single-blinded, single-center, and randomized controlled trial to evaluate the effect of hemostatic agents on the preservation of ovarian reserve and hemostasis during LESS ovarian cystectomy. Patients with unilateral ovarian cyst were randomized to the hemostatic agent and coagulation groups according to the hemostasis method. Afterwards, the patients underwent LESS ovarian cystectomy, and hemostasis was performed after ovarian cyst excision according to the assigned hemostasis method. If hemostasis was not completed within 10 min. After discharge, the patients were followed until 3 months after surgery. We compared the hemoglobin, anti-Müllerian hormone (AMH) levels, and ovarian volumes before surgery, and 2 days, 1 week, and 3 months after surgery (3 M-POST), and the decline ratio between the two groups. The decline ratio of serum AMH levels was greater at 3 M-POST in the coagulation than in the hemostatic agent group (median intention-to-treat [ITT], − 36.7 vs. − 13.3%; per-protocol [PP], − 36.8 vs. − 13.3%; *P* < 0.05). Notably, the difference of the decline ratio of serum AMH levels was only shown in endometriosis patients (median; ITT, − 50.7 vs. − 14.4%; PP, − 50.7% vs. − 14.4%; *P* < 0.05), while there was no difference in non-endometriosis patients. In conclusion, Hemostatic agents may be non-inferior to bipolar coagulation for preserving ovarian reserve and hemostasis during LESS ovarian cystectomy, in particular, for endometriosis patients. (Trial registry: ClinicalTrials.gov Identifier NCT03374397).

## Introduction

Preservation of ovarian reserve after ovarian cystectomy is a critical issue, especially in women of childbearing age. After stripping ovarian cysts, hemorrhage often persists in the parenchymal bed of the ovary, and the degree of ovarian reserve preservation is known to vary depending on the hemostatic methods used^1, 2^. Even though surgical suturing is the primary method for hemostasis during ovarian cystectomy, bipolar coagulation is frequently used since multi-port laparoscopic surgery has become more common^3^, and LESS surgery has also emerged as a growing trend for treating benign gynecologic diseases^4, 5^. Moreover, different types of hemostatic agents showing similar hemostatic effects have been developed and introduced in a clinical setting because bipolar coagulation leads to a decrease in ovarian reserve by tissue damage^6, 7^.

Although relevant randomized controlled trials (RCTs) have reported that hemostatic agents showed better effects for preserving ovarian reserve than bipolar coagulation with no difference in the hemostatic effect during laparoscopic ovarian cystectomy^8, 9^, it is challenging to estimate improvements in ovarian reserve preservation and hemostasis from hemostatic agents used during laparoscopic ovarian cystectomy because of the different types of surgical approaches to hemostasis, and inconsistent assessment of the degree of ovarian reserve preservation.

Thus, we performed this study, called the “PRservation of ovArian reserve and Hemostasis during LESS ovArian cystectomy (PRAHA) trial” to compare the degree of ovarian reserve preservation and the hemostatic effects between bipolar coagulation and oxidized cellulose polymer as a hemostatic agent while minimizing the relevant bias.

## Results

### Participants

**Figure 1:**
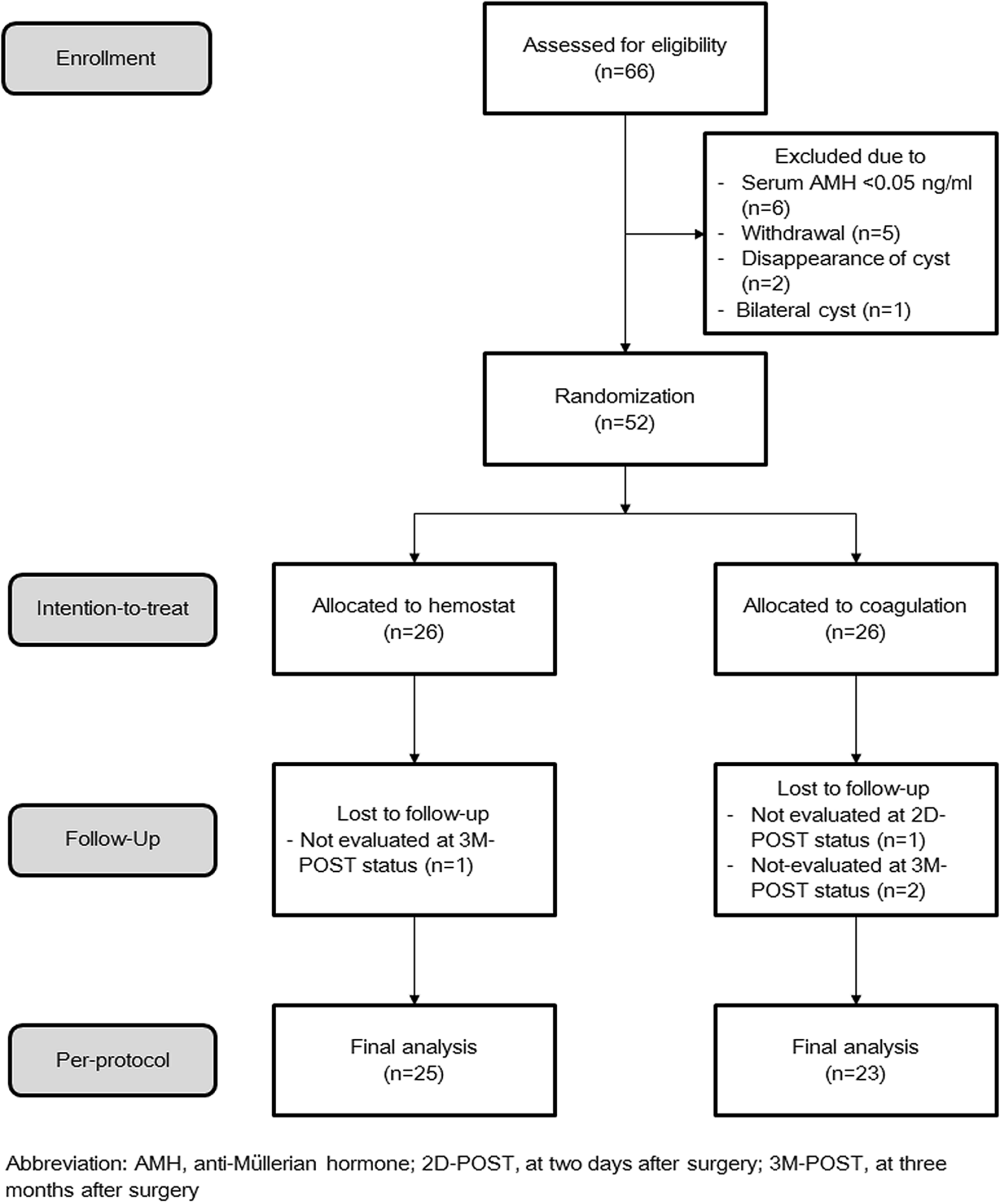
Flowchart based on Consolidated Standards of Reporting Trials.

During this period, 66 patients were assessed for eligibility, and six, five, two, and one patient were excluded due to low anti-Müllerian hormone (AMH) serum levels (< 0.05 ng/mL), refusal to participate, the disappearance of a unilateral ovarian cyst, and the development of bilateral ovarian cysts. Finally, 52 patients were randomly assigned to the coagulation and hemostatic agent groups, and each received planned treatment for ITT analysis. However, four patients were lost to follow-up, and the database of the remaining 48 patients was used for PP analysis (Fig. 1). The clinicopathologic characteristics between the two groups were well-balanced, except for the direction of the ovarian cyst (Table 1).

**Table 1:** Clinicopathologic characteristics.

Characteristics	Hemostatic agent (n = 26, %)	Coagulation (n = 26, %)	*P* value
Age (years)^a^	32.5 ± 6.7	30.3 ± 6.7	0.23
Body mass index (kg/m^2^)^a^	22.6 ± 2.9	21.1 ± 3.7	0.08
Previous abdominal surgery	5 (19.2)	6 (23.1)	1.00
**Direction of ovarian cyst**			< 0.05
Right	17 (65.4)	9 (34.6)	
Left	6 (23.1)	20 (76.9)	
**Histology**			0.45
Endometriosis	12 (46.2)	14 (53.8)	
Mature cystic teratoma	8 (30.8)	10 (38.5)	
Serous cystadenoma	2 (7.7)	0 (0)	
Mucinous cystadenoma	1 (3.8)	0 (0)	
Functional cyst	2 (7.7)	2 (7.7)	
Borderline tumor	1 (3.8)	0 (0)	

^a^Data were shown as mean ± standard deviation.

### Operative outcomes

In terms of operative outcomes, there were no differences in operation time, estimated blood loss, transfusions, hospital stay lengths, complications, and reoperation between the two groups. Moreover, the time for hemostasis and the success rates of hemostasis at 4, 7, and 10 min after surgery did not differ between the two groups (Table 2). In the coagulation group, five patients received additional hemostasis by suturing after 10 min, and among them, four (80%) and one (20%) had endometriosis and mature cystic teratoma, respectively. In the hemostatic agent group, five patients failed to hemostasis within 10 min, and among them, three (60%), one (20%), and one (20%) had endometriosis, a mature cystic teratoma, and a functional cyst, respectively. Ovarian cyst recurrence did not occur during the 3 month follow-up period.

**Table 2:** Operative outcomes.

Parameter	Hemostat agent (n = 26, %)	Coagulation (n = 26, %)	*P* value
Operation time (min)^a^	24.2 ± 11.1	23.3 ± 16.1	.81
Estimated blood loss (mL)^b^	25 (0, 100)	50 (7.5, 50)	.78
**Adhesion**			
No adhesion	21 (80.8)	19 (73.1)	
PCDS	1 (3.8)	3 (11.5)	
Uterus, PCDS	0	3 (11.5)	
Rectum, uterus, PCDS	4 (15.4)	1 (3.8)	
Transfusion	1 (3.8)	0 (0)	1.00
Length of hospital stay (d)^b^	3 (3, 3)	3 (3, 3)	.32
Complication	0 (0)	0 (0)	
Reoperation	0 (0)	0 (0)	
**Hemostasis**			
Time (min)^a^	6.7 ± 4.9	7.2 ± 6.1	.76
Four-min success	11 (42.3)	12 (46.2)	1.00
Seven-min success	15 (57.7)	14 (53.8)	1.00
Additional suturing for incomplete hemostasis after 10 min	5 (19.2)	5 (19.2)	1.00

*PCDS* posterior cul de sac.

^a^Data were shown as mean ± standard deviation.

^b^Data were shown as median with range.

### Hemostasis and preservation of the ovarian reserve

**Figure 2:**
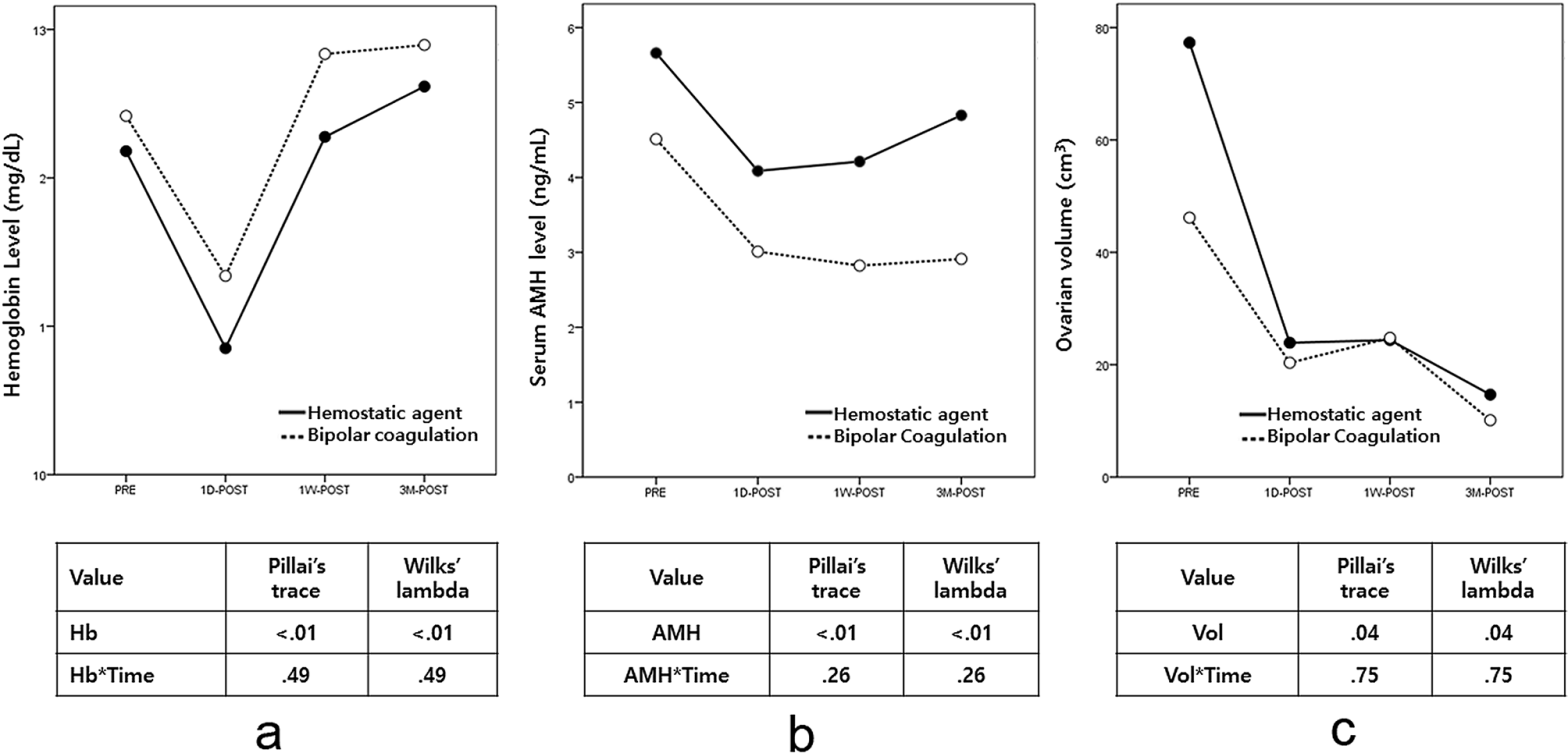
Repeated measure analysis of variance for comparing variables over time (**a**) hemoglobin (Hb) levels (**b**) anti-Müllerian hormone (AMH) levels (**c**) ovarian volume.

Table 3 depicts the comparison of Hb and serum AMH levels, and ovarian volumes at before surgery (PRE), and at 2 days (2D-POST), 1 week (1 W-POST), and 3 months after surgery (3 M-POST), which showed no differences. Figure 2 shows the time-dependent changes in Hb and serum AMH levels and ovarian volumes in the two groups. Hb levels tended to decrease immediately after surgery, and then gradually increased, whereas the serum AMH levels and ovarian volumes tended to decrease over time. However, there were no differences in these trends between the two groups. Furthermore, we evaluated the decline ratio in Hb and serum AMH levels and ovarian volumes between PRE and the other time points. There were no differences in the decline ratio of Hb levels and ovarian volumes between the two groups, whereas the decline ratio of in serum AMH levels was greater 3 M-POST in the coagulation group than in the hemostatic agent group. In particular, the decline ratio of serum AMH levels was greater in the coagulation group than in the hemostatic agent group when only patients with endometriosis were included in the analysis. However, no difference was seen between the two groups when only those with non-endometriosis were analyzed (Table 4).

**Table 3:** Comparison of hemoglobin (Hb) levels and serum anti-Müllerian hormone (AMH) levels and ovarian volume, and the intention-to-treat (ITT) and the per-protocol populations (PP).

Characteristics	ITT			PP		
	Hemostatic agent (n = 26)	Coagulation (n = 26)	*P* value	Hemostatic agent (n = 25)	Coagulation (n = 23)	*P* value
**Hb (mg/dL)**^**a**^						
All						
PRE	12.2 ± 1.7	12.4 ± 1.2	0.76	12.2 ± 1.7	12.4 ± 1.1	0.57
2D-POST	10.9 ± 1.5	11.2 ± 1.2	0.37	10.9 ± 1.5	11.3 ± 1.1	0.21
1 W-POST	12.3 ± 1.4	12.7 ± 1.2	0.25	12.3 ± 1.4	12.8 ± 1.1	0.13
3 M-POST	12.6 ± 1.1	12.9 ± 1.1	0.31	12.6 ± 1.1	12.9 ± 1.1	0.39
Endometriosis						
PRE	11.4 ± 1.8	12.3 ± 1.2	0.15	11.4 ± 1.8	12.4 ± 1.2	0.12
2D-POST	10.5 ± 1.9	11.1 ± 1.2	0.31	10.5 ± 1.9	11.1 ± 1.2	0.30
1 W-POST	11.7 ± 1.6	12.7 ± 1.1	0.08	11.7 ± 1.6	12.8 ± 1	0.06
3 M-POST	12.2 ± 1.3	13 ± 1.1	0.10	12.2 ± 1.3	13 ± 1.1	0.10
Non-endometriosis						
PRE	12.9 ± 1.3	12.6 ± 1.2	0.33	12.9 ± 1.3	12.5 ± 1.1	0.44
2D-POST	11.2 ± 0.9	11.4 ± 1.2	0.77	11.2 ± 0.9	11.6 ± 1	0.34
1 W-POST	12.9 ± 1	12.9 ± 1.3	0.99	12.8 ± 1.1	12.9 ± 1.1	0.86
3 M-POST	13 ± 0.8	12.9 ± 1.1	0.73	13 ± 0.8	12.8 ± 1.1	0.54
**AMH (ng/mL)**^**a**^						
All						
PRE	5.7 ± 5.6	4.6 ± 4.2	0.46	5.7 ± 5.7	4.5 ± 4.3	0.44
2D-POST	4.1 ± 4.3	4.3 ± 4.3	0.30	4.1 ± 4.4	3.0 ± 2.4	0.31
1 W-POST	4.3 ± 4.3	2.8 ± 2.2	0.13	4.2 ± 4.4	2.8 ± 2.4	0.19
3 M-POST	4.8 ± 2.9	6.3 ± 2.9	0.17	4.8 ± 6.3	2.9 ± 2.9	0.19
Endometriosis						
PRE	3.8 ± 3.2	4.4 ± 3.5	0.63	3.8 ± 3.2	3.9 ± 3.1	0.92
2D-POST	2.5 ± 1.9	2.9 ± 2.1	0.64	2.5 ± 2	2.8 ± 2.1	0.78
1 W-POST	2.5 ± 1.9	2.4 ± 1.5	0.85	2.5 ± 2	2.3 ± 1.5	0.82
3 M-POST	2.6 ± 2	2.1 ± 2	0.51	2.6 ± 2	2.1 ± 2	0.51
Non-endometriosis						
PRE	7.2 ± 6.8	4.9 ± 5.1	0.33	7.4 ± 7.1	5.3 ± 5.6	0.45
2D-POST	5.5 ± 5.4	3.3 ± 2.6	0.26	5.5 ± 5.6	3.3 ± 2.8	0.27
1 W-POST	5.8 ± 5.2	3.3 ± 2.9	0.15	5.8 ± 5.5	3.5 ± 3.1	0.24
3 M-POST	6.9 ± 8.1	3.8 ± 3.1	0.26	6.9 ± 8.1	4 ± 3.6	0.31
**Ovarian volume (cm**^**3**^**)**^**b**^						
All						
PRE	25.3 (2.9, 911.7)	23.5 (5.4, 293.5)	0.55	25.1 (2.9, 911.7)	24.6 (5.4, 293.5)	0.89
2D-POST	13.1 (1.2, 217.1)	11.1 (0.6, 149.2)	0.49	12.6 (1.2, 217.1)	11.2 (0.6, 149.2)	0.77
1 W-POST	16.1 (2.1, 190.9)	13.4 (6.4, 258.6)	0.26	15.9 (2.1, 190.9)	13.6 (6.4, 258.6)	0.50
3 M-POST	11 (3.4, 66.4)	9.8 (1.2, 43.7)	0.07	11 (3.4, 66.4)	9.2 (1.2, 43.7)	0.06
Endometriosis						
PRE	19.4 (8.2, 216.5)	16.9 (5.4, 293.5)	0.82	19.4 (8.2, 216.5)	15.3 (5.4, 293.5)	0.65
2D-POST	16.2 (1.2, 217.1)	11.4 (0.6, 149.2)	0.56	16.2 (1.2, 217.1)	11.2 (0.6, 149.2)	0.44
1 W-POST	17 (2.1, 190.9)	10.8 (6.4, 258.6)	0.35	17 (2.1, 190.9)	10.4 (6.4, 258.6)	0.38

*PRE* just before surgery, *2D-POST* at 2 days after surgery, *1 W-POST* at 1 week after surgery, *3 M-POST* at 3 months after surgery.

^a^Data were shown as mean ± standard deviation.

^b^Data were shown as median with range.

**Table 4:** Comparison of decline ratio of hemoglobin (Hb) levels and serum anti-Müllerian hormone (AMH) levels, and ovarian volume in the intention-to-treat (ITT) and the per-protocol populations (PP).

Characteristics	ITT			PP		
	Hemostatic agent (n = 26)	Coagulation (n = 26)	*P* value	Hemostatic agent (n = 25)	Coagulation (n = 23)	P value
**Hb (%)**^**a**^						
All						
Decline ratio 2D^b^	− 10.8 ± 7.6	− 8.6 ± 7.7	0.30	− 10.6 ± 7.7	− 8.5 ± 7.8	0.34
Decline ratio 1W^c^	1.2 ± 7.1	3.3 ± 7.0	0.29	1.3 ± 7.2	3.7 ± 7.3	0.27
Decline ratio 3M^d^	5 ± 13.9	3.9 ± 6.6	0.73	5 ± 13.9	4.1 ± 6.7	0.78
Endometriosis						
Decline ratio 2D^b^	− 8.6 ± 6.7	− 9.2 ± 8.8	0.83	− 8.6 ± 6.7	− 9.7 ± 9	0.73
Decline ratio 1W^c^	2.8 ± 6.4	3.2 ± 6.5	0.87	2.8 ± 6.4	3.7 ± 6.6	0.74
Decline ratio 3M^d^	7.9 ± 16.4	5.1 ± 6.7	0.57	7.9 ± 16.4	5.1 ± 6.7	0.57
Non-endometriosis						
Decline ratio 2D^b^	− 12.7 ± 8	− 7.7 ± 6.3	0.10	− 12.7 ± 8	− 7.7 ± 6.3	0.09
Decline ratio 1W^c^	− 0.1 ± 7.6	3.5 ± 7.8	0.25	− 0.1 ± 7.6	3.5 ± 7.8	0.28
Decline ratio 3M^d^	2.3 ± 11.1	2.5 ± 6.6	0.95	2.3 ± 11.1	2.5 ± 6.6	0.88
**AMH (%)**^**a**^						
All						
Decline ratio 2D^b^	− 27.6 ± 16.3	− 28 ± 25.8	0.94	− 28 ± 16.5	− 28.4 ± 25	0.95
Decline ratio 1W^c^	− 20.5 ± 28.1	− 33.1 ± 29.2	0.12	− 21.1 ± 28.5	− 32.3 ± 29.5	0.19
Decline ratio 3M^d^	− 13.3 ± 34.1	− 36.7 ± 34.3	0.02	− 13.3 ± 34.2	− 36.8 ± 35	0.02
Endometriosis						
Decline ratio 2D^b^	− 27.8 ± 15.2	− 27.1 ± 26.4	0.94	− 27.8 ± 15.2	− 24.9 ± 26.1	0.74
Decline ratio 1W^c^	− 24.6 ± 29.8	− 34.7 ± 32	0.42	− 24.6 ± 29.8	− 31.6 ± 31.1	0.57
Decline ratio 3M^d^	− 14.4 ± 40.5	− 50.7 ± 32.8	0.02	− 14.4 ± 40.5	− 50.7 ± 32.8	0.02
Non-endometriosis						
Decline ratio 2D^b^	− 27.4 ± 17.8	− 29.2 ± 26.2	0.84	− 28.2 ± 18.2	− 33 ± 24.1	0.59
Decline ratio 1W^c^	− 16.9 ± 27.1	− 31.2 ± 26.7	0.19	− 17.9 ± 28	− 33.2 ± 29	0.21
Decline ratio 3M^d^	− 12.2 ± 28.8	− 20.2 ± 29.2	0.51	− 12.2 ± 28.9	− 18.6 ± 30.3	0.61
**Ovarian volume (%)**^**e**^						
All						
Decline ratio 2D^b^	− 45.2 (− 99.3, 154.8)	− 53.7 (− 96.3, 189.5)	0.77	− 41.8 (− 99.3, 154.8)	− 52.9 (− 96.3, 189.5)	0.75
Decline ratio 1W^c^	− 26.9 (− 91.4, 138.1)	− 48.4 (− 97.6, 401.8)	0.74	− 26.6 (− 91.4, 138.1)	− 50.9 (− 97.6, 401.8)	0.91
Decline ratio 3M^d^	− 56.5 (− 98.3, 223.3)	− 51.1 (− 99.6, 123.4)	0.90	− 56.5 (− 98.3, 223.3)	− 52.4 (− 99.6, 123.4)	0.73
Endometriosis						
Decline ratio 2D^b^	− 38.5 (− 85, 40.4)	− 49.5 (− − 96.3, 189.5)	0.84	− 38.5 (− 85, 40.4)	− 46.2 (− 96.3, 189.5)	0.89
Decline ratio 1W^c^	− 24.4 (− 82.6, 17.2)	− 48.5 (− 97.6, 401.8)	1.00	− 24.4 (− 82.6, 17.2)	− 46.1 (− 97.6, 401.8)	0.81
Decline ratio 3M^d^	− 47.7 (− 83.1, 217)	− 47 (− 100. .123.4)	0.85	− 47.7 (− 83.1, 217)	− 47 (− 100, 123.4)	0.85
Non-endometriosis						
Decline ratio 2D^b^	− 63 (− 99.3, 154.8)	− 61.7 (− 87, 56.5)	1.00	− 57.9 (− 99.3, 154.8)	− 61.7 (− 87, 56.5)	0.83
Decline ratio 1W^c^	− 42.1 (− 91.4, 138.1)	− 42.6 (− 78.5, 103)	0.63	− 27.2 (− 91.4, 138.1)	− 58.5 (− 78.5, 103)	0.98
Decline ratio 3M^d^	− 63.1 (− 98.3, 223.3)	− 68.8 (− 96.1, 40)	0.87	− 63.1 (− 98.3, 223.3)	− 73.7 (− 96.1, 39.9)	0.61

^a^Data were shown as mean ± standard deviation.

^b^Defined as (the value at 2 days after surgery—the value just before surgery)/the value just before surgery × 100.

^c^Defined as (the value at 1 week after surgery—the value just before surgery)/the value just before surgery × 100.

^d^Defined as (the value at 3 months after surgery − the value just before surgery)/the value just before surgery × 100.

^e^Data were shown as median with range.

## Discussion

We evaluated the effect of oxidized cellulose polymer as one of the hemostatic agents on the preservation of ovarian reserve and hemostasis in patients who underwent LESS ovarian cystectomies. The hemostatic agent had a hemostatic effect similar to bipolar coagulation and a better effect for preserving ovarian reserve than bipolar coagulation.

Relevant RCTs have reported no difference in the hemostatic effect between bipolar coagulation and hemostatic agents^8–12^. In these studies, the combination of gelatine granules and human thrombin (Floseal; Baxter Healthcare Corporation Fremont, CA, USA) produced a better hemostatic effect than oxidized cellulose polymers^13^ which were mainly used for comparing the hemostatic effect to bipolar coagulation. However, few studies to date have evaluated whether oxidized cellulose polymers, expected to have a less hemostatic effect than the combination of gelatine granules and human thrombin, could have a hemostatic effect similar to bipolar coagulation during laparoscopic ovarian cystectomy.

In terms of the preservation of ovarian reserve, three relevant RCTs emphasized the beneficial effect of the combination of gelatine granules and human thrombin for preserving the remaining ovarian reserve after laparoscopic ovarian cystectomy^8, 10, 11^. In these studies, the preservation effect was calculated to measure the serum AMH levels PRE and 3 M-POST, as in this study. The decline ratio of serum AMH levels ranged from 41.2 to 41.9% in patients treated with bipolar coagulation, whereas they were 15.4–16.1% in those treated with the hemostatic agent. However, these studies could not evaluate the decline ratio in serum AMH levels after unilateral ovarian cystectomy because 13–35% of the patients had bilateral ovarian cysts, which requires further investigation in well-designed RCTs.

Another RCT evaluated the effect of oxidized regenerated cellulose (ORC) on ovarian reserve after laparoscopic cystectomy of endometriosis^14^. In the study, AMH decline ratio at 6 months decreased by 54.1% in the cystectomy-only group, and 45.4% in the cystectomy and ORC apply group. Besides, endometriosis recurrence was significantly higher in the group that applied ORC during the follow-up period up to 30 months. Although the AMH decline ratio was greater in the study than in our study, the hemostasis method was not mentioned in the group that did not apply ORC In our study, follow-up was only performed for up to 3 months, whereas in the study, follow-up was performed for up to 30 months, and it was confirmed that the effect of lowering the recurrence of endometriosis as well as preserving ovarian reserve could be expected by using ORC, which might form a chemical ablation in the ovarian cortex after cystectomy.

In this study, we found that oxidized cellulose polymer showed a similar hemostatic effect as bipolar coagulation during LESS ovarian cystectomies. To the best of our knowledge, this is the first RCT to show a hemostatic effect of oxidized cellulose polymer comparable to bipolar coagulation during LESS ovarian cystectomy. When we consider that the combination of gelatine granules and human thrombin is more expensive than oxidized cellulose polymer, and a diagnosis-related group (DRG) program has been adopted for most of the gynecologic surgeries in our country since July 2013^15^, this finding is significant because oxidized cellulose polymer can be used cost-effectively and safely for hemostasis during LESS ovarian cystectomy.

Moreover, we compared the time-dependent changes in ovarian volumes between the two groups and found no difference in the decline ratio of ovarian volumes despite similar tendencies to decrease after surgery. However, we found a beneficial effect of the hemostatic agent on the preservation of the remaining ovarian reserve compared to bipolar coagulation. In this study, we found a decline in serum AMH levels of 13.3% in the hemostatic agent group, compared to a decline by bipolar coagulation of 36.7%. Above all, we should note that this effect was shown only in patients with endometriosis, and serum AMH levels were declined by 50.7% after bipolar coagulation during LESS ovarian cystectomy. In contrast, there was no difference in the decline ratio between the two treatments in patients with non-endometriosis.

Although thermal damage by bipolar coagulation can further reduce ovarian function compared to hemostatic agents^16^, the experienced gynecologist who performed the LESS ovarian cystectomies made an effort to minimize the removal of healthy ovarian tissue and the use of bipolar coagulation for preserving the remaining ovarian reserve in this study. No differences in the decline ratio of ovarian volume between the two groups support the success of this effort.

Furthermore, this effort could have contributed to the lack of difference in the decline ratio of serum AMH levels between the hemostatic agent and coagulation groups when only patients with non-endometriosis were included in the analysis. The preservation effect by minimizing the use of bipolar coagulation can be supported by previous studies where there was no difference in the decline ratio of serum AMH levels between the hemostatic agent and bipolar coagulation groups, (23 vs. 19%; *P* = 0.47)^9^, and there was no clear association between the remaining ovarian reserve and the number of follicles removed in the specimens^17^.

However, patients with endometriosis showed further decreases in AMH 3 M-POST in terms of decline ratio in the coagulation group. Despite relevant evidence, this finding can be explained by the following hypothesis. Since most patients with endometriosis show pelvic adhesion, we commonly perform adhesiolysis sufficiently from tissues surrounding the lesion for complete cystectomy. During the procedure, the vascular system within the ovarian cortex or surrounding the ovary can be injured, which can result in lower serum AMH levels caused by inadequate blood supply^17^. This hypothesis can be supported by a previous study that showed a lower peak systolic velocity in the ovary subjected to laparoscopic surgery^18^. Furthermore, endometriosis itself or surgery might decrease ovarian reserve than non-endometriotic cyst because the formation of the endometriotic cyst wall is formed by invagination of ovarian cortex comprising ovarian reserve^19^.

Although this study showed the beneficial effect of the hemostatic agent on the preservation of ovarian reserve with a hemostatic effect similar to bipolar coagulation during LESS ovarian cystectomy, the standard method of hemostasis is still suturing, even though there is a relatively long learning curve for laparoscopic suturing. RCTs that compare the hemostatic and preservation efficacy between hemostatic agents and suturing during LESS ovarian cystectomy are needed and performed by experienced gynecologists with surgical proficiency. Moreover, RCTs evaluating the decline ratio of serum AMH levels according to the two hemostatic methods are required for patients with unilateral ovarian endometriosis because the sample size in this study was calculated based on the results of a previous study where various types of ovarian cysts were included^8^.

The strengths of this study are that we enrolled only patients with a unilateral ovarian cyst, and researchers performed supervise for consistency of surgical procedures during the LESS ovarian cystectomies using the same surgical protocol, which minimized the use of bipolar coagulation for preserving ovarian reserve. Moreover, we evaluated ovarian function in terms of serum AMH levels and ovarian volume. As a result, we found a prominent decrease in serum AMH levels using bipolar coagulation in patients with endometriosis. In contrast, the decline ratio of the ovarian volume was similar between the two treatments, suggesting that ovarian volume could not reflect the remaining ovarian reserve after ovarian cystectomy.

The limitation is that we did not evaluate all parameters for more than 3 months after surgery. Regarding that primordial follicle growth may take up to 180 days, at list 6 months follow up after surgery might be sufficient to evaluate ovarian reserve. The relatively small sample size could also act as a bias for interpreting these results despite the well-designed trial. Additionally, the severity of adhesion or impaired vascularity in the enucleated ovary was not evaluated and could have reduced the serum AMH levels, especially in patients with endometriosis. Furthermore, more relevant studies should be conducted for validating these results because a single institution in this study performed the LESS ovarian cystectomies.

## Materials and methods

### Study design

This study was an investigator-initiated, single-blinded, randomized controlled trial conducted at Seoul National University Hospital. The Institutional Review Board of Seoul National University Hospital approved the study (No. 1707-079-869) before study initiation and registered at ClinicalTrials.gov on December 15, 2017 (No. NCT03374397). We obtained informed consent from all participants, and study was performed according to the relevant guidelines and regulations the Institutional Review Board of Seoul National University Hospital.

### Participants

We enrolled patients with benign ovarian diseases consecutively from December 2017 to February 2019 based on the following eligibility criteria: age ≥ 18 and ≤ 45 years; unilateral benign ovarian cysts confirmed by pelvic ultrasonography; a regular menstrual period between 21 and 45 days; preoperative serum AMH levels of ≥ 0.50 ng/mL; planned LESS ovarian cystectomy; American Society of Anesthesiologists (ASA) physical status classification 1–2; and written informed consent. The exclusion criteria were as follows: suspicious ovarian malignancy shown by imaging studies; bilateral ovarian disease; preoperative serum AMH levels of < 0.50 ng/mL; planned multi-port laparoscopic ovarian cystectomy; pregnancy or breastfeeding; comorbid endocrine disease, such as thyroid function abnormality, hyperprolactinemia, or Cushing’s disease; and history of hormonal therapy within 3 months. A simple ultrasound-based rule was used for evaluation of the ovarian cyst^20^. Benign rules included unilocular, size of solid component less than 7 mm, acoustic shadows, smooth multilocular cyst less than 10 cm, and no Doppler flow, and malignant rules included irregular margin, ascites, more than three papillary structure, and presence of Doppler flow.

### Sample size calculation

The sample size was calculated based on a relevant RCT^8^, where the decline ratio of AMH at 3 months after surgery was 41.2% (interquartile range 17.2–54.5%) and 16.1% (interquartile range 8.3–44.7%) in the bipolar coagulation and hemostatic agent groups, respectively. The mean value of the difference between the two groups was 25.1%, and the standard deviation estimated from the assumption of normal distribution was 27.7% and 27% for each group. When the sample size was based on a non-inferiority margin of 30%, reflecting an acceptable difference between the two groups, the estimated sample size was 52 patients to obtain 80% power and the two-sided significance levels of 5%, considering that 10% of the target number of patients would be eliminated.

### Randomization

All patients were randomly assigned to the coagulation and hemostatic agent groups at a 1: 1 ratio after signing the informed consent form. A third-party gynecologist (AS) created and managed the randomization table using a web-based program. Randomization table was created by simple calculation without blockings. Then, AS managed the sequence without the other gynecologists knowing. The randomization table was implemented with silver paper, so that the next assignment cannot be recognized. The randomization results were released to investigators immediately before surgery, and blinding of patients was maintained until the end of the study.

### Surgical procedure

All patients underwent LESS ovarian cystectomy by the single gynecologist (HSK) with more than 100 cases per year of 10 years of experience, and all surgical procedures were monitored by authors SJP and NL for consistency. The patient was placed in the Trendelenburg position after general anesthesia, and a Glove Port A (Medtech Inframed Corp., Seoul, Republic of Korea) was inserted through the umbilicus. If unilateral ovarian cysts adhered to the surrounding tissues, they were mobilized from the surrounding tissues, and then the ovarian cortex was incised with monopolar scissor on the opposite side of the mesovarium longitudinally. The cleavage plane between the ovarian cortex and the cyst wall was identified, and cyst enucleation was performed by pulling the cyst wall in a direction opposite to the ovarian cortex. After completing cyst enucleation, bipolar coagulation was applied for patients in the coagulation group. In contrast, one piece of non-woven SurgiGuard, 10.2 × 10.2 cm (Hanmi Pharm. Co., Ltd., Seoul, Republic of Korea) was applied in the inner bleeding area of the ovarian cortex for those in the hemostatic agent group. If hemostasis failed within 10 min, suturing was performed in the coagulation group, whereas bipolar coagulation, and suturing if needed, were conducted in the hemostatic agent group. After confirming the lack of bleeding, the peritoneal cavity was irrigated with normal saline. All patients were discharged 2 days after surgery.

### Endpoints

In previous relevant studies, serum AMH levels, follicular stimulating hormone, the antral follicular count, and ovarian volume were used as markers for evaluating the remaining ovarian reserve after ovarian cystectomy. Among them, serum AMH levels are known to be stable during regular menstrual cycles and decline with advanced age, reflecting the remaining ovarian reserve^21–23^. In this study, we considered the decline ratio of serum AMH levels 3 months after surgery as the primary endpoint, which was defined as 100 × (the serum AMH level 3 months after surgery minus the serum AMH level just before surgery)/the serum level of AMH just before surgery (%), based on a relevant trial^8^.

The secondary endpoints were operative outcomes including operation time, the estimated blood loss, transfusions, length of hospital stay, complications, reoperation, time for hemostasis, and the success rates of hemostasis at 4, 7, and 10 min. Moreover, we compared the Hb levels (mg/dL) and the serum AMH levels (ng/mL), and ovarian volumes (cm^3^) just before surgery (PRE), and at 2 days (2D-POST), 1 week (1 W-POST), and 3 months after surgery (3 M-POST), and the decline ratio between the two groups. The ovarian volumes were estimated as follows: volume (cm^3^) = maximal sagittal diameter (length, cm) × maximal coronal diameter (width, cm) × maximal transverse diameter (depth, cm) × 0.5233 on pelvic ultrasonography^24^.

### Statistical analysis

We performed a statistical analysis of both the intention-to-treat (ITT) and the per-protocol populations (PP). The continuous variables between the coagulation and hemostatic agent groups were compared by Student’s t or the Mann–Whitney U test, and the categorical variables were analyzed using Fisher’s exact or the χ^2^ test. Repeated measure analysis of variance (ANOVA), including Pillai’s Trace, and Wilks’ Lambda were used to analyze the outcomes according to the time points. All P-values of < 0.05 were considered statistically significant. The statistical analyses were conducted using SPSS 20.0 (SPSS, Inc., Chicago, IL, USA).

## Conclusions

Oxidized cellulose polymer as a hemostatic agent may be effective for hemostasis and preserving ovarian reserve during LESS ovarian cystectomy compared to bipolar coagulation. Moreover, the use of hemostatic agents in patients of reproductive age suspected to have ovarian endometriosis should be considered to preserve ovarian reserve instead of bipolar coagulation.

## Data Availability

The datasets generated during and/or analyzed during the current study are not publicly available because the informed consent did not include an explanation about public data sharing plan, but are available from the corresponding author on reasonable request.
